# Genome sequence data and properties of *Bifidobacterium bifidum* strain ICIS-504 isolated from multispecies bifidobacterial community

**DOI:** 10.1016/j.dib.2022.108672

**Published:** 2022-10-14

**Authors:** Sergey V. Andryuschenko, Elena V. Ivanova, Natalia B. Perunova, Oleg V. Bukharin, Irina A. Zdvizhkova

**Affiliations:** Institute for Cellular and Intracellular Symbiosis, Ural Branch of RAS, Pionerskaya st., 11, Orenburg 460000, Russian Federation

**Keywords:** WGS, Human residential bifidobacterium, Microbiocoenosis, Cytokines, Response regulator, Coiled-coil region

## Abstract

This report presents the data on the draft genome sequence of *Bifidobacterium bifidum* strain ICIS-504. The strain, isolated from the intestine of a 41-year-old healthy woman is a member of community consist of four strains of three bifidobacterial species: *B. longum, B. bifidum* and *B. breve*. Annotation of the genome sequence revealed as high similarity with deposited strains as the unique duplication in the functional region of the only AmiR-family response regulator gene. The draft genome sequence data of *B. bifidum* strain ICIS-504 is available under the accession nos. JAJJPE000000000.1, PRJNA776132 and SAMN22746550 for NCBI Genome, Bioproject and Biosample databases, respectively.

**Specifications Table**

**Table:** 

Subject	‘Microbiology’
Specific subject area	Microbial genomics of beneficial bacteria
Type of data	Genome assembly, predicted genes and annotation
How data were acquired	Whole genome sequencing of fragment libraries with Illumina MiSeq platform, following de novo genomic assembly
Data format	FASTA format for genome sequences GenBank format for genome annotations
Parameters for data collection	The genomic DNA extraction, fragment library preparation, Illumina sequencing, de novo assembly and annotation procedures
Description of data collection	The genomic DNA extraction was performed using the standard phenol-chloroform method; fragment library was prepared by using Illumina, San Diego, CA Kit. The library was sequenced in a 2 × 300-nucleotide run using the MiSeq reagent kit version 3 and MiSeq desktop sequencer (Illumina). The reads were quality trimmed using the sliding window mode of the Trimmomatic program. De novo assembly of draft genome sequences was performed using SPAdes version 3.10.1 (St. Petersburg genome assembler).
Data source location	The culture of strain B. *bifidum* ICIS-504 is deposited in Institute for cellular and intracellular symbiosis of Ural Division of RAS
Data accessibility	The draft genome sequence data of strain Bifidobacterium bifidum strain ICIS-504 is available in GenBank database under the accession number JAJJPE000000000.1: (https://www.ncbi.nlm.nih.gov/nuccore/NZ_JAJJPE000000000.1). The BioProject database ID of the sequenced strain is PRJNA776132 (https://www.ncbi.nlm.nih.gov/bioproject/PRJNA776132). The sequence read archive of this project is available under the ID SRP346303 (https://trace.ncbi.nlm.nih.gov/Traces/sra/?study=SRP346303). Repository name: Mendeley Data Data identification number: vtxknw7n3y Direct URL to data: https://data.mendeley.com/drafts/vtxknw7n3y/2
Related research article	S.V. Andryuschenko, E.V. Ivanova, N.B. Perunova, O.V. Bukharin. Genome Sequence and Biochemical Properties of Bifidobacterium longum Strain ICIS-505, Isolated from the Intestine of a Healthy Woman. Microbiol Resour Announc. 2019 Aug 15; 8 (33). pii: e00491-19. 10.1128/MRA.00491-19.

## Value of the Data

• Presented strain is a member of multispecies community of human intestinal residential bifidobacteria.
• The described immunoregulatory properties of the presented strain will make it possible to clarify the adaptive potential of *B. bifidum* bacteria in the multispecies consortium of indigenous bacteria of the human intestine.
• High similarity with deposited genomes of *B. bifidum* strains combined with the unique duplication in the functional region of the only AmiR-family response regulator gene makes the presented strain a valuable object for further functional studies.

## 1 Data

The case of four strains of bifidobacteria isolated at once was of particular interest to us. We determined a number of phenotypic properties in the isolated strains and sequenced two strains: B. longum ICIS-505 [1] and B. bifidum ICIS-504, the results of which we present in this communication with an emphasis on the most variable determinants important for bifidobacteria adaptability in the biotope: the set of sortase-dependent fimbriae [2] and two-component signaling systems [3].

The sequenced genome of Bifidobacterium bifidum strain ICIS-504 has a size of 2,250,442 bp (62.7 G+C content) and consist of 95 contigs (N50 = 311,191). The annotation revealed 1,947 gene sequences, including 1,826 proteins, 3 complete and 3 partial rRNA genes, 54 tRNA genes, 3 noncoding RNA genes and 58 pseudogenes.

Sequencing of the strain genome and annotation of the sequences obtained showed unambiguous belonging of the strain to the B. bifidum species and high similarity of its genome sequence with the previously sequenced strain B. bifidum MRI 1 (Institute of Fundamental Science, Pohang University of Science and Technology, Korea, NCBI Reference Sequence Index: NZ_CP018757.1). The strains are characterized by an average number of sortase-dependent fimbriae determinants (4 genes with an LPxTG domain) and a small number of genes of two-component signaling systems: 5 serine-threonine protein kinases, 8 histidine kinases, and 13 response regulators.

However, in the genome of B. bifidum ICIS-504 both genes of one of the two-component regulatory systems remained identical with the previously sequenced probiotic strains of this species: B. bifidum ICIS-202 and B. bifidum 791: both the histidine kinase receptor gene WP_041775364.1 at position 179 retained a serine residue, and the response regulator gene WP_003812704.1 at position 48 retained a valine residue. Both loci are located within the respective functional domains of the BaeS and OmpR families

At the same time, we found known differences in the gene sequences of another histidine kinase and three response regulators of the B. bifidum ICIS-504 genome from both B. bifidum strain MRI 1 and the ICIS-202/791 probiotic strains, as well as one unique difference within the sequence of the functional domain of the AmiR family response regulator (Table 1).

**Table 1:** Sequence differences in annotated genes of two-component regulatory systems of sequenced strains.

	Differences from homologous peptide products of strains	
Genes of the strain B. bifidum ICIS-504	B. bifidum PRI 1	B. bifidum ICIS-202/791
WP_047271810.1 – BaeS family histidine kinase	deletion of 62 AA from the N-terminus	absence of 8 amino acid substitutions
WP_047270947.1 – CitB family response regulator	deletion of 30 AA from the N-terminus	retaining of VQ at 38-39 position
WP_229941019.1 CitB family response regulator	deletion of 12 AA from the N-terminus	retaining of Arg 12 residue
WP_229940945.1 – AmiR family response regulator	Duplication of the LKKAEEK heptad repeat from 184 position	

Analysis of the amino acid sequence WP_229940945.1 of the AmiR family response regulator showed that the heptadic repeat duplication LKKAEEK, unique among the deposited genomes, is located in the central region of the helix, which provides the formation of a functional dimer [4]. At the same time, it has been shown that the length of this region is a conserved trait and is probably reflected in the function of the resulting polypeptide [5].

B. bifidum culture ICIS-504 produced mainly acetic acid (C2), with propionic (C3), isobutyric (IC4), butyric (C4), and valerian (C5) acids registered in trace amounts. At the same time, the level of acetic acid production was lower than the average values determined by us in other 20 strains of this bifidobacteria species (Table 2).

**Table 2:** Phenotypic properties of B. bifidum strain ICIS-504 in comparison with other B. bifidum strains (N=20).

	Production of SCFAs, mol/L				
Strain	C2	C3	IC4	C4	C5
*B. bifidum* ICIS-504	7,0 ± 0,6	0,07 ± 0,06	0,03 ± 0,015	0,05 ± 0,01	0,013 ± 0,002

20 *B. bifidum* strains	11,9 ± 6,9	0,23 ± 0,18	0,06 ± 0,04	0,06 ± 0,02	0,033 ± 0,03

A study of the effect of B. bifidum ICIS-504 metabolites on the level of cytokines secreted by human PBMCs showed a marked increase in interleukin-10 (IL-10) production: 3-fold or more in comparison with the baseline cytokine secretion; the concentration of interleukin-1 receptor antagonist (RaIL-1) was 1.5-fold higher on average. B. longum ICIS-505 metabolites had no significant effect on the secretion of the above anti-inflammatory cytokines by PBMC. The suppressive effect of microbial metabolites of both *B. bifidum* ICIS-504 and *B. longum* ICIS-505 supernatants was observed on the secretion of proinflammatory cytokines: tumor necrosis factor alpha (TNF-α) and interleukin-17 (IL-17), by PBMCs (Table 3). Both strains had no significant effect on interferon gamma (IFN-γ) levels.

**Table 3:** Spontaneous production of cytokines by human peripheral blood mononuclear cells under the influence of metabolites of the studied strains of Bifidobacteria.

	PBMCs cytokine production, pg/ml			
Strain	TNF-α	IL-17	IL-10	RaIL-1
Сontrol	42,6 ± 2,3	140,5 ± 8,6	50,0 ± 4,4	102,5 ± 11,4
*B. bifidum* ICIS-504	9,1 ± 0,1	32,4 ± 4,5	157,8 ± 28,2	153,1 ± 21,3
*B. longum* ICIS-505	17,8 ± 0,7	35,1 ± 3,7	54,2 ± 3,8	105,0 ± 8,3

## 2 Materials and Methods

The studied strains were cultured in 4 ml of Schadler's broth (HiMedia Laboratories Pvt. Limited) for 48 h in an atmosphere of 0.6% oxygen and 5% carbon dioxide at 37 C, as described in [6]. DNA isolation, library preparation, sequencing, as well as genome assembly and annotation were performed in full accordance with the technique described in our previous work. Bifidobacteria production of SCFAs was determined by gas chromatography: cultured samples were centrifuged at 13600 g for 15 min, and 50 μl of 98% sulfuric acid (Panreac, Germany) was added to 500 μl of the supernatant. Extraction of volatile fatty acids from the samples was performed in 750 μl isobutyl alcohol (Sigma-Aldrich, USA) and the process was repeated twice. Acetate extraction was performed in a GC-2010 Plus gas-liquid chromatograph (Shimadzu, Japan) equipped with a flame ionization detector with a HP-FFAP capillary column (Agilent Technologies, USA), diameter 0.32 mm, length 50 meters. Evaporator temperature was 240 °C; temperature program for capillary column: 0 min - 70 °C, 10 min - 160 °C, 5 min - 180 °C and 25 min - 240 °C; detector temperature - 260 °C. Helium was used as a carrier gas, the carrier gas speed was 21 cm/s. The peak area concentrations were calculated using GCsolution software (Shimadzu, Japan). To assess the immunoregulatory activity of bifidobacteria, we determined the ability of mononuclear cells isolated and incubated according to the method of previous work [6] to produce the indicated cytokines in concentrations measured by enzyme immunoassay using the method described in [7].

## Ethics Statements

This work did not contain human subjects, animals, cell lines or endangered species.

## Declaration of Competing Interest

The authors declare that they have no known competing financial interests or personal relationships that could have appeared to influence the work reported in this paper.

## Data Availability

WGS data of Bifidobacterium bifidum ICIS-504 (Original data) (Mendeley Data).
